# On the Comparative Advantages of the Cooling and Heating Treatment of Gouty and Rheumatic Inflammation

**Published:** 1817-10

**Authors:** 


*For the London Medical and Physical Journal.*

### *On the comparative Advantages of the Cooling and Heating Treatment of Gouty and Rheumatic Inflammation;* by Dr. Kinglake.

THE part which I have taken in advocating the cooling treatment of gouty and rheumatic inflammation has ever been induced by a conscientious persuasion that a most important alleviation of human suffering was likely to be effected by it. Ample experience had fully satisfied every ambiguity in my mind on the general expediency and safety of the treatment. It was not proposed on a partial and doubtful survey of its effects, but on a comprehensive and clear estimate of its influence. It was utterly impossible to resist the demonstrative testimony which reiterated proofs of its salutary efficacy had afforded. It would therefore ill become me to retract an iota of my conviction of its beneficial powers; but it is rather incumbent on me to offer it the further defence and recommendation which additional experience is daily affording. No misrepresentation nor obloquy can affect its real merits. Whatever illiberal invective may be employed to decry its just reputation, it will be found to stand with unshaken firmness against every species of sinister endeavour. Bottomed, as the principle of the treatment is, on the nature of things, it cannot be successfully assailed; it will yield to no declamation, to no hypothetical artifices, whilst positive facts will prove it to be incontrovertibly well founded.

I have been led into this unnecessary eulogium on the practical advantages of the cooling remedy, by two recent instances of the pernicious effects of the opposite treatment, —in one of which, death closed a protracted period of acute suffering; in the other, a disabled state of the affected joints, with but very little prospect of their ever becoming again useful, is the lamented result. The subjects of both those cases were young, and the duration of the respective complaints was for several weeks. The whole period was passed in exhausting irritation, certainly not in *salutary pain.* Judging from analogy, founded on an extensive review of actual occurrences, it is but just to conclude that an anti-stimulant and an evacuant mode of treatment, early and unremittedly applied, would, in the instances under consideration, have at once broken the force, shortened the continuance, and subdued the affection soon enough to have obviated the mortality and decrepitude that resulted from its prolonged existence. Inflammatory affections of every variety, as to form and situation, require to be repressed and extinguished by sedative influence, which, to speak intelligibly, consists in diminishing immoderate excitement. Whatever may have induced the inflammatory state, or however it may be denominated when produced, that morbid condition of vital action should be directly and speedily overcome. This cannot be effected by half-measures, by indirect and circuitous processes, that have rather the name than the reality of being at all efficient. Observation and patience are essential requisites in medical practice, but these will be unavailing if they lead not to the decisive adoption of adequate modes of cure. Some diseases wear themselves out sooner than others: the spontaneous exhaustion of gouty inflammation is a very tardy process, arising from the peculiar structure of the parts in which the diseased action is seated; hence it would be expecting too much to calculate on its dissipating of its own own accord in any time that might not either seriously involve the structure of the diseased parts in irremediable derangement, or even the general health itself in deep, complicated, and irreparable disease. To foster and encourage gouty or any other species of morbid excitement for an indefinite period, seems to be refining too much on the remedial powers of disease to be at all admissible in correct medical reasoning. That one diseased action will, occasionally, overcome another, may be true; but that any description of disease is preferable to a state of health, one would imagine is hardly conceivable by the most visionary theorist. The momentous fact is, that disease is an active condition of vital power, to which no precise bounds can be prescribed: its progress and issue, therefore, are always too anxious a concern to admit of reposing any confidence in its salutary nature and tendency.

Popular prejudice has been excited against topical cold to an inflamed surface, by contrasting it with the warmth that has been usually judged indispensably necessary. Contrasts naturally awaken a dread of the opposite effect, and beget the state of feeling that is alive to the slightest cause of alarm. A proneness to misinterpret is an inseparable associate of fear, and, under such groundless apprehensions, the utmost terror, confusion, and obscurity are apt to prevail. The morbidly excited state of an inflamed part is a condition of vital action that generates excessive heat; and it is undeniably true that heat is the most direct and efficient stimulus that can be applied to the inherent irritability of animal life. It is, then, the excessive influence of this exciting power, whether it acts as a cause or an effect, as a substance or a property, that should be the leading object of attention in endeavouring to effect a cure. How then is this to be accomplished but by the sedative or negative management of stimulant power, so as to bring its degree or intensity down to the limits of healthy action? This can only be rationally attempted by suitably diminishing its force, and it can only be actually effected by its adequate abstraction. Internal stimulants, and the external detention and envelopment of heat, cannot be consistently regarded as appropriate means of cure; but the avoidance of both the one and the other affords a reasonable chance of benefit.

The timid prejudices of the public on this subject have not been liberally met: instead of having been removed by rational explanation, they have been rather strengthened and confirmed by medical alarmists and medical routinists, who have either warranted themselves in encouraging the groundless apprehensions that prevail, or of conniving at them from an an utter indifference to the correction of error and the advancement of truth in medical science. Unprofessional patients, like other persons, can only reason from what they know: they pretend not to canvass hypothetical views of disease; they are for the most part unacquainted with the theory of disease, but they are not wholly ignorant of its practical notices and injunctions. When left, therefore, to their own feelings, they often conceive, and act in correct unison with common sense, and in a way that renders them too decided in their conviction to be afterwards ever shook by any endeavour to detach them from their firm persuasion. There are many who possess this description of faith in the cooling treatment of gout, and who will not be shifted from it by any contrivance that may aim at depriving them of its advantage. Others are less experienced, and, of course, less decided: to these it may be right to propose measures of conciliation, that will, perhaps, ultimately enable them to become its unreserved and zealous advocates.

To that part of the public, therefore, that may be yet terrified by unjust suspicion of the noxious effects of topical cold in gouty inflammation, it might be expedient to insure them the eventual benefit of the remedy through the less revolting medium of grateful warmth. So much of direct and complete relief cannot, perhaps, be furnished in this circuitous mode of remedy, yet much important aid may be rendered, and that too in consonance with, or at least without shocking, the popular prejudice on the subject. To such patients, therefore, it would be right to advise an incessant moistening of the parts suffering under gouty or rheumatic inflammation with a tepid fluid. This may be done either by a sponge or more effectually by cloths wetted in it, renewing them as often as they become dry. The fluid should be aqueous, or, for the purpose of rendering it more evaporable, one-sixteenth of either æther or brandy may be conjoined with it. The drying of the part will be the detachment of stimulant heat, and the cooling effects of reduced temperature will be felt on the inflamed surface.

The refrigerating influence produced by incessantly moistening the inflamed part with a tepid fluid, and leaving it to dry by evaporation, will be very powerful. Although the effect may not be so immediate as from positive cold, yet much of its advantages may be thus eventually realised. The compromise, therefore, is allowable in as far as it involves no dereliction of principle, and may be likely, under circumstances of occurring fear and delusion, to be employed in a degree commensurate with the intention of cure. What, in this way, is conceded to mode, is preserved in reality, and and the practical benefit of diminished heat in gouty inflammation may be much extended by the proposed method of effecting it. As powerful auxiliaries in promoting the desired relief, opening medicine should be taken every other day. Animal food should be avoided, and a slight farinaceous diet adopted, to the entire exclusion of every species of fermented liquor. These anti-stimulant means will soon avail in reducing and finally overcoming the inflammatory excitement, and in protecting the system at large from the exhausting and often disorganising effects of lengthened and unalleviated pain.

*Taunton; July* 28, 1817.

